# A Higher Frequency of Circulating IL-22^+^CD4^+^ T Cells in Chinese Patients with Newly Diagnosed Hashimoto’s Thyroiditis

**DOI:** 10.1371/journal.pone.0084545

**Published:** 2014-01-03

**Authors:** Hui Guo, Di Peng, Xi-Ge Yang, Ye Wang, Bing-Chuan Xu, Jin-Song Ni, Wei Meng, Yan-Fang Jiang

**Affiliations:** 1 Key Laboratory for Zoonosis Research, Department of Central Laboratory, The First Hospital, Jilin University, Changchun, China; 2 Department of Endocrinology, The Second Part of the First Hospital, Jilin University, Changchun, China; 3 Department of Anesthesiology, The First Hospital, Jilin University, Changchun, China; 4 Department of Pathology, The Second Part of the First Hospital, Jilin University, Changchun, China; 5 Department of Thyroid Surgery, The First Hospital, Jilin University, Changchun, China; Northwestern University Feinberg School of Medicine, United States of America

## Abstract

**Background:**

IL-22 and IL-17A are implicated in the pathogenesis of autoimmune diseases. However, the role of IL-22^+^ and IL-17A^+^ CD4^+^ T cells in the pathogenesis of Hashimoto’s thyroiditis (HT) is not fully understood. This study investigates serum IL-22 and IL-17A levels and determines the frequency of circulating IL-22^+^ CD4^+^ T cells in HT patients to understand their roles in the pathogenesis of HT.

**Methods:**

The levels of serum IL-22, IL-17A and IFN-γ and the frequency of circulating IL-22^+^CD4^+^ and IL-17A^+^CD4^+^ T cells in 17 HT patients and 17 healthy controls (HC) were determined by enzyme-linked immunosorbent assay (ELISA) and flow cytometry. The levels of serum free triiodothyronine (FT4), free thyroxine (FT3), thyroid stimulating hormone (TSH), anti-thyroid peroxidase (TPO) and anti-thyroglobulin antibodies (TgAb) by chemiluminescent enzyme immunoassay and radioimmunoassay.

**Results:**

The percentages of circulating IL-22^+^CD4^+^ and IL-17^+^CD4^+^ T cells (p<0.0001, p<0.0001) and the levels of serum IL-22, IL-17A and IFN-γ (p<0.0001, p<0.0001, p = 0.0210) in the HT patients were significantly higher than that in the HC. The percentages of IL-22^+^CD4^+^ T cells were positively correlated with Th17 cells (r = 0.8815, p<0.0001) and IL-17A^+^IL-22^+^CD4^+^ T cells (r = 0.8914, p<0.0001), but were negatively correlated with Th1 cells (r = −0.6110, p<0.0092) in the HT patients. The percentages of Th22 cells, Th17 cells and IL-17A^+^IL-22^+^CD4^+^ T cells were negatively correlated with the levels of serum TSH in the HT patients (r = −0.8402, p<0.0001; r = −0.8589, p<0.0001; r = −0.8289 p<0.0001, respectively).

**Conclusions:**

A higher frequency of circulating IL-22^+^CD4^+^ and IL-17A^+^CD4^+^ T cells may be associated with the development of HT in Chinese patients.

## Introduction

Hashimoto’s thyroiditis (HT) is an organ-specific autoimmune disease and is characterized by lymphocytic infiltrates within the thyroid glands [1], [2]. Autoreactive T cells, natural killer (NK) cells, autoantibodies as well as pro-inflammatory cytokines contribute to the destruction of thyroid cells, leading the development of HT [3], [4]. Currently, the pathogenesis of HT is not fully understood. While previous studies have shown that antigen-specific CD8^+^ T cell-mediated cytotoxicity and autoantibody-dependent cytotoxicity are crucial in the pathogenesis of HT, they are regulated by different functional CD4^+^ T cells [5]. Therefore, understanding the regulation of different functional CD4^+^ T cells will be of great significance.

Upon antigen stimulation, naïve CD4^+^ T cells activate and differentiate into different subsets of functional T cells, such as IFN-γ-secreting Th1, IL-4-secreting Th2, IL-17A-secreting Th17 and IL-22-secreting Th22 as well as IL-10- and TGFβ1-secreting Tregs [6]. Previous studies have shown that Th1 cells are major players in the pathogenesis of HT, while Tregs negatively regulate its pathogenesis [3], [7]. Recent studies indicated that HT patients have a higher frequency of circulating Th17 cells and elevated levels of serum IL-17A [3], [8]. However, it is unclear how Th1 and Th17 cells contribute to the early process of HT.

Activated CD4^+^ T cells differentiate into Th22 cells, which are driven by the aryl hydrocarbon receptor (AHR) transcription factor and positively regulated by IL-6 and TNFα as well as plasmacytoid dendritic cells [9]. Previous studies indicated that Th22 cells secrete IL-22, but not IL-17A and IFN-γ [9]–[11]. A recent study suggests that IL-22 cells are also secreted by some IL-17A^+^CD4^+^ T cells [10]. However, it is unclear whether IL-17A^+^IL-22^+^CD4^+^ T cells exist in HT patients and what the relationship among Th22, Th17 and IL-17A^+^IL-22^+^ T cells is. Functionally, Th22 cells have been suggested to participate into the pathogenesis of autoimmune diseases, such as ankylosing spondylitis (AS), inflammatory bowel disease (IBD), psoriasis and rheumatoid arthritis (RA) [6], [10]–[12]. However, there is a lack of study on the frequency of spontaneously activated Th22 cells in HT patients and whether these cells can infiltrate into the thyroid gland tissue. Furthermore, the relationship between the frequency of Th22 cells, the values of the thyroid functional measures and the levels of serum autoantibodies in HT patients is not understood.

In this study, we characterized the frequency of peripheral blood Th22, Th17 and Th1 cells using flow cytometry analysis and measured the levels of serum IL-22, IL-17, IFN-γ, autoantibodies, free triiodothyronine (FT4), free thyroxine (FT3), thyroid stimulating hormone (TSH), using ELISA and radioimmunoassay in 17 Chinese patients with newly-diagnosed HT and 17 gender- and age-matched healthy controls (HC). The levels of IL-22 and IL-17A expression in the thyroid gland tissues from 15 HT patients, who underwent a surgical resection of the thyroid nodules, were determined by immunohistochemistry. We analyzed the potential correlation of the frequency of Th22, Th17 and Th1 cells with the clinical parameters in HT patients.

## Materials and Methods

### Ethics Statement

The experimental protocol was established in accordance with the guidelines of the Declaration of Helsinki and was approved by Human Ethics Committee of Jilin University.

### Study Subjects

Seventeen patients with newly-diagnosed HT were recruited from the inpatient service of the First Hospital of Jilin University, Changchun, China between January and September 2012. Seventeen gender- and age-matched healthy controls (HC) were recruited from the Health Examination Center of the same hospital. Individual HT patients were diagnosed based on the particular ultrasonographic pattern in the thyroid glands, the abnormal serum levels of anti-thyroid peroxidase antibodies (TPOAb) and/or anti-thyroglobulin antibodies (TgAb). There were 15 patients receiving surgical resection of the thyroid nodules and their thyroid tissue samples were examined pathologically to confirm the diagnosis of HT. The levels of IL-22 and IL-17A expression in individual tissue samples were determined by immunohisotochemistry. Subjects were excluded if she/he had a history of and current chronic respiratory diseases, smoking, neuromuscular disease, narcolepsy, transient ischemic attack, chronic heart failure, craniofacial abnormalities, autoimmune hepatitis, type 1 diabetes (T1DM), multiple sclerosis (MS), RA, IBD, alcoholic abuse or sedative drug user, current pregnancy, other endocrinological diseases and recent infection. Written informed consent was obtained from each participant. The demographic and clinical data of the participants were obtained from hospital records and reviewed by experienced physicians. The demographic and clinical characteristics of the participants are summarized in Table 1.

**Table 1:** The demographic and clinical characteristics of the participants.

	Healthy controls (n = 17)	HT patients (n = 17)
Gender (M/F)	2/15	1/16
Age (year) median(range)	43 (30–56)	48 (37–66)
TSH (µIU/mL)	2.00 (0.29–2.99)	3.04 (0.31–4.58)
FT4 (pmol/L)	14.78 (12.89–17.21)	16.41 (14.36–21.98)
FT3 (pmol/L)	4.56 (3.31–4.97)	4.86 (3.45–6.58)
TPO-Ab (IU/mL)	17.00 (8–24)	157.0 (7.45–600.00)*
Tg-Ab (IU/mL)	50.00 (32–80)	270.3 (31.39–652.70)*
WBC (×10^9^/L)	5.5 (4.78–6.83)	5.8 (4.7–8.23)
Lymphocytes (×10^9^/L)	2.30 (1.40–3.30)	2.31 (1.45–3.33)

p<0.01 vs. HC.

Data shown are the real case numbers or median (range) of each group. The clinical normal range for TSH: 0.27–4.2 µIU/mL; FT4∶12.0–22.0 pg/mL; FT3∶3.1–8.6 pg/mL; TPOAb: <35.0 IU/mL; TgAb: <115.0 IU/mL.

### Laboratory Examinations

Venous blood samples were obtained from the participants for full blood cell counts and other laboratory tests. The levels of serum FT3, FT4, TSH, TGAb and TPOAb were determined using chemiluminescent enzyme immunoassay (Ortho-Clinical Diagnostics, Raritan, NJ, USA) and radioimmunoassay (Cosmic Corporation, Tokyo, Japan) in accordance with the manufacturers’ instructions.

### Isolation and Stimulation of PBMCs

Fasting peripheral venous blood samples were collected from individual participants and peripheral blood mononuclear cells (PBMCs) were isolated by density-gradient centrifugation using Ficoll-Paque Plus (Amersham Biosciences, Little Chalfont, UK). PBMCs at 10^6^ cells per mL were stimulated in duplicates with phorbol 12-myristate 13-acetate (PMA, 50 ng/ml; Sigma, St. Louis, MO, USA) and ionomycin (1 µg/mL; Sigma, St. Louis, MO, USA) in RPMI 1640 medium (Invitrogen, Carlsbad, CA, USA) containing 10% human AB-type of serum and 55 µM 2-Mercaptoethanol (2-ME) at 37°C in a humidified incubator with 5% carbon dioxide (CO_2_) for 2 hours. The cells are then cultured for another 4 hours in the presence of Brefeldin A (BFA, 0.5 µg/mL; Sigma). The negative control cells were cultured in RPMI 1640 medium (Invitrogen) alone.

### Flow Cytometry Analysis

The stimulated PBMCs were harvested and stained with allophycocyanin (APC)-conjugated anti-CD4 and (APC-H7)-conjugated anti-CD3, fixed and permeabilized with the permeabilization/fixation solution (eBiosciences, San Diego, USA), followed by staining with PE-conjugated anti-IL-22 (R&D Systems, Minneapolis, MN, USA), FITC-conjugated anti-IL-17 (BD Pharmingen, San Diego, CA, USA), and PE-Cy7-conjugated anti-IFN-γ (BD Pharmingen, San Diego, CA, USA). The frequency of CD4^+^IFN-γ^–^IL17A^–^IL-22^+^ (Th22), CD4^+^IFN-γ^–^IL17A^+^IL-22^−^ (Th17), CD4^+^IFN-γ^−^IL17A^+^IL-22^+^(Th17/Th22) and CD4^+^IFN-γ^+^ (Th1) T cells in the samples was determined by flow cytometry analysis on a BD FACSAria II (Becton Dickinson) using FlowJo 7.6.2 software.

### ELISA

The IL-22, IL-17, and IFN-γ serum levels in individual participants were determined using cytokine ELISA kits in accordance with the manufacturers’ instruction (R&D Systems, Minneapolis, MN, USA). The detection limit for IL-22, IL-17, and IFN-γ was 0.1 pg/mL.

### Immunohistochemistry

The thyroid tissues were fixed in 10% formalin and paraffin-embedded. The thyroid tissue sections (5.0 µm) were deparaffinized, rehydrated, and treated with 3% H_2_O_2_ to inactivate endogenous peroxidase. The sections were blocked with 5% fat-free dry milk in Tris-Buffered Saline and Tween 20 (TBST) for 30 min and incubated with rabbit anti-human anti-IL-22, rabbit anti-human IL-17 antibodies or rabbit control sera (1∶500 dilutions; Zymed Laboratories, San Diego, USA) overnight at 4°C. After being washed, the bound antibodies were detected using biotinylated goat anti-rabbit IgG at room temperature for 30 min and incubated with avidin-biotinylated horseradish peroxidase (HRP) complex using the Histostain-Plus Kit (ZYMED) and diaminobenzidine tetrahydrochloride (DAB; Biosynthesis Biotechnology, USA). The sections were then counterstained with Mayer’s hematoxylin (Bioss, Beijing, China). The intensity of immunostaining in the nuclei and cytoplasm of thyroid follicular cells was quantified using Image-pro plus image analysis software (Media Cybernetics, Georgia,USA).

### Statistical Analysis

All data are expressed using either individual mean values or median and range of each group of subjects. The difference between two groups was analyzed by the Kruskal-Wallis H nonparametric test. The potential correlations between variables were evaluated by using Spearman’s rank correlation test in SPSS 19.0 for Windows (SPSS, Chicago, IL, USA). A two-sided P value of <0.05 was considered statistically significant.

## Results

### A Higher Frequency of Pro-inflammatory CD4^+^ T Cells in Patients with Newly-diagnosed HT

To determine the potential role of different subsets of CD4^+^ T cells, 17 patients with newly-diagnosed HT and 17 HC were recruited. There was no significant difference in the distribution of age and gender between the HT patients and HC. There was also no significant difference in the levels of serum TSH, FT4 and FT3 and the numbers of white blood cells (WBC) and lymphocytes between these groups. However, the levels of serum APOAb and TgAb in HT patients were significantly higher than that in the HC (p<0.01). These data indicated that those patients were at the euthyroid state.

**Figure 1:**
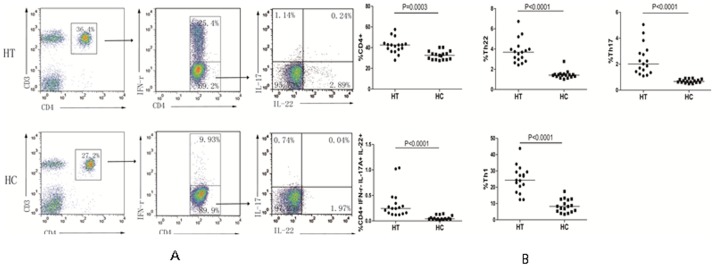
Flow cytometry analysis of the frequency of different subsets of CD4+ T cells. PBMCs were isolated from individual participants and stimulated with PMA and ionomycin for six hours in the presence of BFA. The cells were stained with fluorescent antibodies against CD3 and CD4, fixed and permeabilized, followed by intracellular staining with anti-IFN-γ, anti-IL-17A and anti-IL-22. The cells were gated on CD3+CD4+ T cells and the percentages of IFN-γ^−^IL-17A^−^IL-22^+^, IFN-γ^−^IL-22^−^IL-17A^+^ IFN-γ^−^IL-17A^+^IL-22^+^, IFN-γ^+^IL-17A^−^IL-22^−^ and IFN-γ^+^CD4^+^T cells in CD3+CD4+ T cells were determined. Data are representative dot plots and the percentages of different subsets of CD4+ T cells in the HT patients and HC. The horizontal lines indicate the median values.

Aberrant activation of autoreactive T cells is associated with the development of HT. To characterize the frequency of different subsets of CD4^+^ T cells, PBMCs were prepared from individual participants and stimulated with PMA and ionomycin. The cells were stained with APC-anti-CD4 and intracellularly stained with antibodies against the different cytokines studied. The frequency of CD4^+,^ CD4^+^IFN-γ^−^IL-17A^−^IL-22^+^ (Th22), CD4^+^IFN-γ^−^IL-17A^+^IL-22^−^ (Th17), CD4^+^IFN-γ^−^IL-17A^+^IL-22^+^ (Th17/Th22) and CD4^+^IFN-γ^+^ (Th1) T cells was characterized by flow cytometry (Fig. 1). We found that the percentages of CD4^+^ T, Th22, Th17, Th17/Th22, and Th1 cells in HT patients were significantly higher than that in HC (P<0.001 for every measure). Hence, a higher frequency of pro-inflammatory CD4^+^ T cells existed in patients with newly-diagnosed HT.

### The Relationship among Different Subsets of CD4^+^ T Cells in the HT Patients

**Figure 2:**
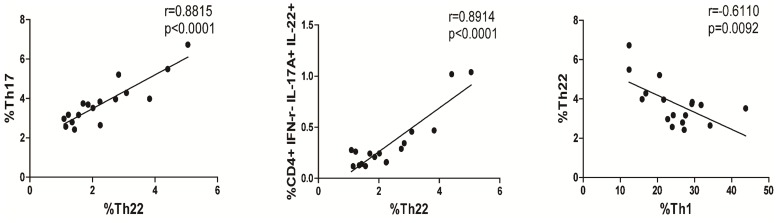
Correlation between percentages of different subsets of CD4+ T cells in HT patients. The potential correlations among the different subsets of CD4+ T cells were analyzed by Spearman’s rank correlation test. Data shown are the mean values of individual patient (n = 17).

To understand the relationship among different subsets of CD4^+^ T cells, we performed correlation analyses. We found that the percentages of Th22 cells were positively correlated with the percentages of Th17 cells (r = 0.8815 p<0.0001, Fig. 2) and Th17/Th22 cells (r = 0.8914, p<0.0001, Fig. 2), but negatively correlated with the percentages of Th1 cells in newly-diagnosed HT patients (r = −0.6110, p = 0.0092, Fig. 2).

### The Percentages of Circulating Th22, Th17 and Th17/Th22 Cells are Correlated Negatively with Serum TSH Levels in Newly-diagnosed HT Patients

**Figure 3:**
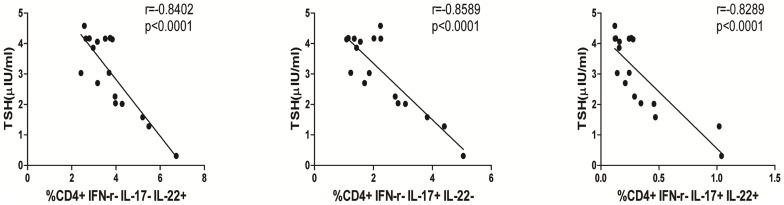
Correlation between percentages of different subsets of CD4+ T cells and levels of serum TSH in newly diagnosed HT patients. The potential correlations between the levels of serum TSH and the percentages of Th22, Th17 cells in the HT patients were analyzed by Spearman’s rank correlation test. Data shown are the mean values of individual patients (n = 17).

TSH produced from thyrotrope cells in the anterior pituitary gland stimulates the thyroid glands to produce FT4 and FT3. Although newly-diagnosed HT patients can have normal levels of serum TSH, FT3 and FT4, they should have elevated levels of serum TgAb and TPOAb. We further analyzed the relationship between the percentages of Th22, Th17, Th22/Th17 or Th1 cells and the levels of serum TSH, FT3, FT4, TgAb and TPOAb in newly-diagnosed HT patients. We found that the levels of serum TSH were negatively correlated with the percentages of Th22, Th17 and Th22/Th17 cells (r = −0.8402, p<0.0001; r = −0.8589, p<0.0001; r = −0.8289 p<0.0001 respectively, Fig. 3). However, there was no statistically significant association between other indicators of thyroid function and the percentages of different subsets of CD4^+^ T cells tested in the HT patients (data not shown).

### Elevated Levels of Serum IL-22, IL-17 and IFN-γ in Newly Diagnosed HT Patients

**Figure 4:**
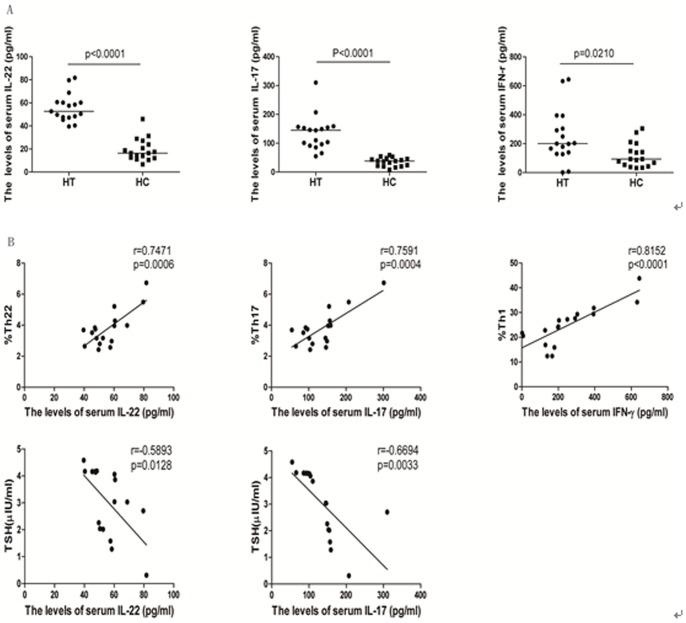
The levels of serum IL-22, IL-17A, and IFN-γ in each subject. The levels of serum IL-22, IL-17A, and IFN-γ in each subject were measured by ELISA and the potential association of the concentrations of serum cytokines with the percentages of corresponding cells or the levels of serum TSH was analyzed by Spearman’s rank correlation test. Data are expressed as the mean values of individual subjects (n = 17 for each group) from four separate experiments. A. ELISA analysis of the levels of serum cytokines; B. Correlation analysis. The levels of serum IL-17A were not significantly correlated with the percentages of Th17/Th22 cells in those patients (data not shown). The levels of serum IL17A and IL-22 were not correlated with the values of other measures tested in the HT patients (data not shown).

Different subsets of T cells secrete their specific cytokines. By measuring the levels of serum IL-22, IL-17 and IFN-γ in each study participant by ELISA, we found that the levels of serum IL-22, IL-17 and IFN-γ in the HT patients were significantly higher than that in HC (p<0.001, p<0.001, p = 0.021, respectively; Fig. 4A). Further analysis revealed that IL-22 serum levels were positively correlated with the percentages of Th22 (r = 0.7417, p = 0.0006; Fig. 4B); IL-17 serum levels were positively correlated with the percentages of Th17 (r = 0.7591, p = 0.0004; Fig. 4B); and serum levels of IFN-γ were positively correlated with the percentages of Th1 cells (r = 0.8152, p<0.0001, Fig. 4B) in newly diagnosed HT patients. More importantly, the levels of serum IL-22 and IL-17 were also negatively correlated with the levels of serum TSH in those patients (r = −0.5893, p = 0.0128; r = −0.6694, p = 0.0033, Fig. 4B).

### Elevated Level of IL-22 is Detected in the Thyroid Tissue Lesions of Newly-diagnosed HT Patients

**Figure 5:**
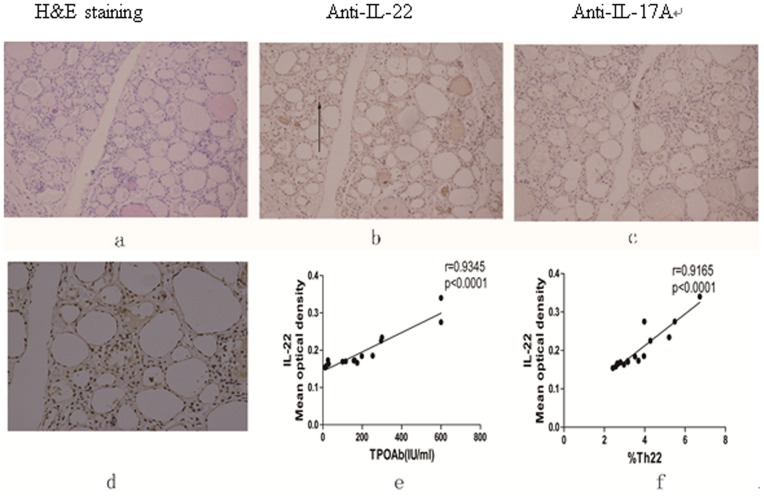
Immunohistochemistry analysis of IL-17A and IL-22 in the thyroid tissues of newly diagnosed HT patients. The thyroid tissue samples were obtained from 15 patients, who were subjected to a surgical resection of the thyroid nodules and the thyroid tissue sections were characterized by H&E staining and immunohistochemistry analysis of IL-17A and IL-22 expression. The mean optical density (MOD) of immunostaining was quantified. The potential correlation of MOD values for anti-IL-22 staining with the levels of serum TPOAb and the percentages of Th22 cells in the HT patients was analyzed. Data shown are representative images and expressed as the mean values of individual patients. A. HE staining of the thyroid tissue section (magnification ×200); B. Immunohistochemical detection of IL-22 in the thyroid tissues of a HT patient (magnification ×200). C. Immunohistochemical detection of IL-17A (magnification ×200). D. Immunohistochemical staining of anti-IL-22 (magnification ×400). Correlation analyses of IL-22 mean optical density with E. TPOAb and F. %Th22.

Finally, we examined the levels of IL-22 and IL-17 expression in the thyroid glands by immunohistochemistry. We detected moderate levels of IL-22 and a little IL-17A mainly in thyroid follicular cells of the thyroid glands from the HT patients (Fig. 5A). Further analysis revealed that the mean optical density (MOD) of anti-IL-22 staining was positively correlated with the levels of serum TPOAb (r = 0.9345, p<0.0001) and the percentages of circulating Th22 cells (r = 0.9165, p<0.0001) in the HT patients (Fig. 5B). However, there was no statistically significant association of the intensity of anti-IL-22 staining in the thyroid sections with other indicators of thyroid function and different subsets of CD4^+^ T cells tested in the HT patients (data not shown).

## Discussion

CD4+ T cells, such as Th1 and Th17 cells, play a complicated and important role in inflammatory and autoimmune diseases [13]–[16]. We have also recently found that, besides Th1 and Th17 cells, Th22 cells also contribute to the development of autoimmune thyroiditis in humans [17]. In this study, we examined the frequency of circulating Th22, Th17 and Th1 cells in patients with newly diagnosed HT and HC. A significantly higher frequency of Th1, Th17, Th22 and CD4^+^IFN-γ^−^IL-17A^+^IL-22^+^ T cells and significantly higher levels of serum IFN-γ, IL-17A and IL-22 existed in newly-diagnosed HT patients, related to that in the HC. These data extended our previous findings that a higher frequency of Th17 and Th22 in new-onset GD patients [17], suggesting that HT and GD may share with a similarity in the pathogenic process. Our findings extended previous findings [16], [18], [19], supporting the notion that multiple types of pro-inflammatory T cells are involved in the development of HT. However, our results were in disagreement with a previous observation of decreased Th1 responses in HT patients [4]. The difference may stem from varying stages of patients studied by different groups.

Engagement of T cell receptor (TCR) on naive CD4+ T cells activates the cells, which subsequently differentiate into different subsets of pro-inflammatory CD4+ T cells, including Th1, Th17 and Th22. The differentiation of activated CD4+ T cells depends on the expression of specific transcription factors and cytokine environment. T-bet determines Th1 differentiation, while RORγt and AHR transcription factor specify Th17 and Th22 differentiation, respectively. Interestingly, the percentages of Th22 cells were found to be positively correlated with the percentages of Th17 and CD4^+^IFN-γ^−^IL-17A^+^IL-22^+^ T cells, but negatively correlated with the percentages of Th1 cells in newly diagnosed HT patients. These data suggest that Th17 and Th22 cells may share similar pathways and activation environment during the HT pathogenesis and CD4^+^IFN-γ^−^IL-17A^+^IL-22^+^ T cells may be intermeddlers of differentiated Th17 and Th22. Indeed, interleukin-6 (IL-6) is a critical cytokine for Th17 differentiation and positively regulates Th22 development [11]. More importantly, we found that the percentages of circulating CD4^+^IFN-γ^−^IL-17A^+^IL-22^+^, Th17, and Th22 cells and the level of serum IL-17A and IL-22 were negatively correlated with the levels of serum TSH in those patients, respectively. Serum TSH level is a valuable measure for the screening, diagnosis and monitoring of primary hypothyroidism. It is well known that normal levels of TFH are usually detected in patients with HT at the early disease process due to compensated secretion of TSH from the thyroid. However, may patients will develop hypothyroidism and display lower levels of TSH at the late stage of the disease process. Our results indicated that significantly higher percentages of circulating IL-22^+^CD4^+^ and IL-17^+^CD4^+^ T cells were detected in the HT patients than in the HC, and the percentages of Th22 cells, Th17 cells and IL-17A^+^IL-22^+^CD4^+^ T cells were negatively correlated with the levels of serum TSH in the HT patients. These data suggest that significantly higher percentages of Th22, Th17 and Th17/Th22 may be associated with the pathogenic process of HT. Although the levels of serum TSH in the HT patients were not significantly different from that in the HC the inverse correlation between the percentages of inflammatory T cells and the levels of TSH in the HT patients may suggest that strong inflammatory T cell responses may eventually result in the development of hypothyroidism in the HT patients during the disease progression. It is possible that both Th22 and Th17 cells synergistically contribute to the development of HT in patients, leading to the development of hypothyroidism in the HT patients.

During the pathogenesis of HT, pro-inflammatory T cells infiltrate the thyroid gland tissues and chronically destroy thyroid gland function. Previous studies have shown that inflammatory cells are present in the thyroid glands. In this study, we characterized the presence of IL-17A and IL-22 in the thyroid glands by immunohistochemistry. Although we failed to detect IL-17A, we did detect positive staining of anti-IL-22 in the thyroid gland tissue sections of newly diagnosed HT patients. More importantly, the MOD values of anti-IL-22 staining were positively correlated with the percentages of circulating Th22 cells and the levels of serum TPOAb in those patients. Although the levels of serum TPOAb are not clearly correlated with HT severity, abnormal levels of serum TPOAb are one of the biomarkers for HT diagnosis in the clinic. The significant correlation between the MOD values of anti-IL-22 staining in the thyroid glands, the levels of serum TPOAb and the percentages of circulating Th22 cells in these patients suggest that the percentages of Th22 cells and the levels of serum IL-22 may serve as supplementary biomarkers for the diagnosis of HT.

In summary, our data indicated higher frequency of circulating Th22 and Th17 cells and higher levels of serum IL-22 and II-17A in patients with HT. The percentages of circulating Th22 and Th17 cells and the levels of serum IL-22 and IL-17A were negatively correlated with the levels of serum TSH in those patients. More interestingly, we detected anti-IL22 staining in the thyroid gland tissue sections, with MOD values positively correlated with the levels of serum TPOAb and the percentages of circulating Th22 cells. Our data suggest that Th22 cells and secreted IL-22 may participate in the early disease process of HT and the percentages of circulating Th22 cells and the levels of serum IL-22 may serve as supplementary biomarkers for the diagnosis of HT. We recognized that our study is limited by the small sample size, and that there was a lack of longitudinally functional studies of these inflammatory cells with multiple measures during the pathogenesis of HT as well as a lack of TSH-stimulation blocking antibody detection. Therefore, further longitudinal studies of the function of these inflammatory cells during the pathogenesis of HT in a bigger population are warranted.
